# Inclusion of *Yucca schidigera* extract into finishing diets: impacts on ruminal, physiological, and productive responses of feedlot cattle

**DOI:** 10.1093/tas/txad071

**Published:** 2023-06-30

**Authors:** Shea J Mackey, Reinaldo F Cooke, Autumn T Pickett, Luis F D Batista, Egleu D M Mendes, Michael J Rincker, Eduardo A Colombo

**Affiliations:** Department of Animal Science, Texas A&M University, College Station, TX 77845, USA; Department of Animal Science, Texas A&M University, College Station, TX 77845, USA; Department of Animal Science, Texas A&M University, College Station, TX 77845, USA; Department of Animal Science, Texas A&M University, College Station, TX 77845, USA; Department of Animal Science, Texas A&M University, College Station, TX 77845, USA; Distributors Processing, Inc., Porterville, CA 93257, USA; Department of Animal Science, Texas A&M University, College Station, TX 77845, USA

**Keywords:** feedlot cattle, performance, physiology, rumen, *Yucca schidigera*

## Abstract

This experiment compared ruminal, physiological, and productive responses of feedlot cattle receiving *Yucca schidigera* extract to replace or fed in conjunction with monensin + tylosin. Angus-influenced steers (*n* = 120) were ranked by body weight (**BW**; 315 ± 3 kg) and allocated to 4 groups of 30 steers each. Groups were housed in 1 of 4 drylot pens (30 × 12 m) equipped with GrowSafe feeding systems (4 bunks/pen) during the experiment (day −14 to slaughter). On day 0, groups were randomly assigned to receive a diet containing (2 × 2 factorial): 1) no inclusion or inclusion of monensin + tylosin (360 mg and 90 mg/steer daily, respectively) and 2) no inclusion or inclusion of *Y. schidigera* extract (4 g/steer daily). Steers were slaughtered in 3 groups balanced by treatment combination (36 steers on day 114, 36 steers on day 142, and 48 steers on day 169). Blood was sampled on days 0, 28, 56, and 84, and the day before shipping to slaughter. On day 41, eight rumen-cannulated heifers (BW = 590 ± 15 kg) were housed with steers (1 pair/pen). Pairs rotated among groups every 21 d, resulting in a replicated 4 × 4 Latin square (*n* = 8/treatment combination) with 14-d washout intervals. Heifers were sampled for blood and rumen fluid at the beginning and end of each 21-d period. Monensin + tylosin inclusion decreased (*P* < 0.01) feed intake and improved (*P* = 0.02) feed efficiency of steers, but did not alter (*P* ≥ 0.17) steer BW gain or carcass merit traits. Inclusion of *Y. schidigera* extract did not impact (*P* ≥ 0.30) steer performance and carcass characteristics. Plasma glucose, insulin, insulin-like growth factor-I, and urea-N concentrations were not affected (*P* ≥ 0.16) by monensin + tylosin, nor by *Y. schidigera* extract inclusion in steers and heifers. Ruminal pH in heifers was increased (*P* = 0.04) by monensin + tylosin, and also by (*P* = 0.03) *Y. schidigera* extract inclusion. Rumen fluid viscosity was reduced (*P* = 0.04) by *Y. schidigera* extract, and rumen protozoa count was increased (*P* < 0.01) by monensin + tylosin inclusion. The proportion of propionate in the ruminal fluid was increased (*P* = 0.04) by monensin + tylosin, and tended (*P* = 0.07) to be increased by *Y. schidigera* extract inclusion. Hence, *Y. schidigera* extract yielded similar improvements in rumen fermentation compared with monensin + tylosin, but without increasing performance and carcass quality of finishing cattle. No complimentary effects were observed when combining all these additives into the finishing diet.

## Introduction

Feedlot diets are often enriched with feed additives, such as the antimicrobials monensin and tylosin, to improve production efficiency while mitigating digestive disorders in cattle (Samuelson et al., 2016). With the increasing regulations regarding the use of feed-grade antimicrobials (US Food and Drug Administration, 2015), novel dietary strategies to improve health and performance of feedlot cattle are warranted. An example is supplementing cattle with *Yucca schidigera* extract, which is a plant-derived feed ingredient that contains approximately 18% saponins (dry matter basis; **DM**) and shown to improve ruminal environment and fermentation responses (Singer et al., 2008; McMurphy et al., 2014a).

Our research group recently evaluated the inclusion of *Y. schidigera* extract into high-grain diets that did not contain monensin nor tylosin (Sousa et al., 2019; Rett et al., 2020). Sousa et al. (2019) reported that supplementing the *Y. schidigera* extract at 2 g/d (as-fed basis) increased feed efficiency and average daily gain (**ADG**) in feedlot-receiving cattle. Rett et al. (2020) supplemented *Y. schidigera* extract (0, 1, 2, or 4 g/heifer daily; as-fed basis) to heifers receiving a grain-based bloat provocative diet. These authors reported that *Y. schidigera* extract supplementation increased rumen propionate concentration and decreased acetate:propionate ratio. Moreover, heifer ADG and feed efficiency increased linearly with the inclusion of *Y. schidigera* extract. Others have also reported that *Y. schidigera* extract enhanced ruminal fermentation by inhibiting growth of protozoa and bacteria associated with liver abscess such as *Streptococcus* spp. (Wallace et al., 1994; Nagaraja and Lechtenberg, 2007).

The productive and biological impacts of including *Y. schidigera* extract into feedlot diets containing monensin and tylosin, or to substitute these feed-grade antimicrobials still warrant investigation. Johnson et al. (2015) supplemented *Y. schidigera* extract at 1 g/d to finishing steers consuming a high-grain diet containing monensin, and did not report any improvements in steer performance and carcass merit traits. Based on the results from Rett et al. (2020), we hypothesized that *Y. schidigera* extract fed at 4 g/d is an alternative to feed-grade antimicrobials in improving rumen function and performance of finishing cattle. Therefore, this experiment compared ruminal, physiological, and productive responses of feedlot cattle receiving *Y. schidigera* extract to replace or fed in conjunction with monensin and tylosin.

## Materials and Methods

This experiment was conducted at the Texas A&M – Beef Cattle Systems (College Station, TX). All animals were cared for in accordance with acceptable practices and experimental protocols reviewed and approved by the Texas A&M AgriLife Research, Agriculture Animal Care and Use Committee (#2021-0308).

### Animals and Treatments

#### Finishing steers.

One hundred and twenty Angus-influenced steers were assigned to this experiment. On day −14, steers were weighed (315 ± 3 kg), implanted with Revalor-XS (Merck Animal Health, Kenilworth, NJ), fitted with a half-duplex radio frequency identification ear tag (Allflex USA Inc., Dallas, TX), vaccinated against respiratory (Vista Once SQ; Merck Animal Health) and clostridial pathogens (Cavalry 9; Merck Animal Health), and received an anthelmintic (SafeGuard; Merck Animal Health). Steers were ranked by body weight (**BW**) and allocated to four groups of 30 steers/group, which were housed in 1 of 4 drylot pens (30 × 12 m) equipped with GrowSafe automated feeding systems (Model 6000E; GrowSafe Systems Ltd, Airdrie, AB Canada; 4 bunks/pen) from day −14 to the end of the experiment.

Groups were randomly assigned on day 0 to receive a corn-based total-mixed ration (**TMR**; Table 1) containing, in a 2 × 2 factorial arrangement of treatments: 1) no inclusion or inclusion of monensin + tylosin (360 mg/steer daily from Rumensin 90 and 90 mg/steer daily from Tylan 40; Elanco Animal Health, Greenfield, IN) and 2) no inclusion or inclusion of *Y. schidigera* extract (4 g/steer daily, as-fed basis; Micro-Aid Feed Grade Concentrate; DPI Global, Porterville, CA). The dosage of monensin and tylosin was selected according to the manufacturer’s recommendation for finishing cattle, whereas the *Y. schidigera* extract dosage was based on the treatment that yielded the greatest ADG and feed efficiency in Rett et al. (2020). Steers had free-choice access to water and a TMR (offered daily at 0800 h), which was fed from day −16 to −1 without the inclusion of treatments. Groups rotated across drylot pens every 14 d to account for any potential pen effects, and were slaughtered at a commercial facility (STX Beef; Corpus Christi, TX) in three groups according to their BW. Each group contained an equivalent number of steers from each treatment combination (36 steers shipped on day 113, 36 steers shipped on day 141, and 48 steers shipped on day 169).

**Table 1.** Composition and nutritional profile of total mixed rations (**TMR**) that included *Yucca schidigera* extract (**YS**), monensin + tylosin (**MT**), a combination of MT and YS, or no feed additives (**CON**)^1^

	Step 1				Step 2				Finishing			
Item	CON	YS	MT	MT + YS	CON	YS	MT	MT + YS	CON	YS	MT	MT + YS
Composition, % dry matter												
Rolled corn	46.0	46.0	46.0	46.0	54.1	54.1	54.1	54.1	61.3	61.3	61.3	61.3
DDGs	22.8	17.6	22.8	17.6	22.8	17.6	22.8	17.4	22.7	18.9	22.7	18.7
Sorghum stalk	22.7	22.7	22.7	22.7	14.8	14.8	14.8	14.8	7.9	7.9	7.9	7.9
Molasses	5.6	5.6	5.6	5.6	6.0	6.0	6.0	6.0	6.2	6.2	6.2	6.2
DDGs + YS mix^2^	0.0	5.2	0.0	5.2	0.0	5.2	0.0	5.4	0.0	3.8	0.0	4.0
Mineral mix A^3^	2.9	2.9	0.0	0.0	2.3	2.3	0.0	0.0	1.9	1.9	0.0	0.0
Mineral mix B^4^	0.0	0.0	2.9	2.9	0.0	0.0	2.3	2.3	0.0	0.0	1.9	1.9
Nutritional profile,^5^ dry matter												
Dry matter, %	84.5				85.3				83.7			
NEm, Mcal/kg	1.76				1.85				1.90			
NEg_,_ Mcal/kg	1.15				1.21				1.26			
ADF, %	15.1				12.6				8.9			
NDF, %	28.1				23.8				17.9			
CP, %	11.8				12.3				13.0			
Starch, %	33.5				38.3				46.6			
Ether extract, %	5.11				5.74				5.00			

^1^Step 1 = days 0 to 6; Step 2 = days 7 to 13; Finishing = days 14 to slaughter. From days −16 to −1, steers were offered an TMR containing (as-fed) 34% rolled corn, 22% dried distillers’ grains (**DDGs**), 34% sorghum stalks, 7.5% molasses, and 2.5% mineral mix A.

^2^DDGs added YS (Micro-Aid Feed Grade Concentrate; DPI Global, Porterville, CA) at 0.744% (as-fed basis).

^3^Containing 20% Ca, 0.02% P, 20% NaCl, 0.26% K, 0.16% Mg, 0.048% Cu, 0.002% Se, 0.560% Zn, 0.150% Mn, 220,000 IU/kg of vitamin A, 19,800 IU/kg of vitamin D3, and 3,500 IU/kg of vitamin E (Anipro Xtraperformance Feeds, College Station, TX).

^4^Mineral mix A with the inclusion (as-fed basis) of sodium monensin (Rumensin 90; Elanco Animal Health, Greenfield, IN) at 0.80% and tylosin (Tylan 40; Elanco Animal Health) at 0.44%.

^5^Based on CON diet analyzed via wet chemistry procedures (Dairy One Forage Lab, Ithaca, NY) and equations described by Cooke et al. (2023). NEm, net energy for maintenance; NEg, net energy for gain; ADF, acid detergent fiber; NDF, neutral detergent fiber; CP, crude protein.

#### Rumen-cannulated heifers.

On day 41 of the experiment, eight rumen-cannulated Angus-influenced heifers (initial BW = 590 ± 15 kg) were divided into four pairs in a manner that all pairs had equivalent mean BW. From days 41 to 54, heifers were maintained in a single group and received the finishing TMR (Table 1) without the inclusion of treatments (at 2.5% of BW; as-fed basis). On day 55, all heifers were fitted with a half-duplex radio frequency identification ear tag (Allflex USA Inc.), and each pair was housed with one group of finishing steers in pens equipped with GrowSafe bunks (1 pair/drylot pen) as in Colombo et al. (2022). Pairs rotated among groups receiving different treatment combinations on 21-d periods, resulting in a replicated 4 × 4 Latin square design arrangement (*n* = 8 heifers/treatment combination). Between periods, heifers were managed in a single group receiving the finishing TMR (at 2.5% of BW; Table 1) without the inclusion of treatments for 14 d as a washout interval.

### Sampling

Samples of the TMR that did not include monensin + tylosin nor *Y. schidigera* extract were collected every other week, pooled across weeks, and analyzed for nutrient content via wet chemistry (Dairy One Forage Laboratory, Ithaca, NY). Calculations for net energy for maintenance and gain used equations from the NASEM (2016). Nutritional profile of the TMR is described in Table 1. All cattle were observed daily for bloat (Meyer and Bartley, 1972) and respiratory disease signs according to the DART system (Zoetis Florham Park, NJ) as in Sousa et al. (2019). No incidence of these diseases nor other causes of morbidity and mortality were observed during the entire experimental period.

#### Finishing steers.

Shrunk BW (after 16-h of feed and water restriction) was recorded from all steers on day −8. Unshrunk BW was also recorded (before the TMR feeding; 0700 h) from all steers on days 0, 28, 56, 84, and 112 of the experiment, and from steers that were still under experimental procedures (not slaughtered) on days 140 and 168. Shrunk BW from day −8 was added a 4% shrink and considered initial BW (321 ± 3 kg). Another full BW was recorded from steers at the time of shipping to slaughter (days 113, 141, and 169), which was averaged with BW recorded the day before and considered final BW. ADG was calculated using initial and final BW. Individual feed intake was evaluated daily from day −14 to slaughter using the GrowSafe 6000E software (GrowSafe System Ltd) as in Parsons et al. (2020), whereas intake from days −7 to −1 was averaged for each steer and considered baseline intake. Ad libitum feed intake was ensured by providing 110% of the dietary intake recorded from each pen in the previous day. Inclusion of monensin + tylosin and *Y. schidigera* extract was adjusted every 7 d according to feed intake of the previous week to ensure that cattle received the designed dosages (Table 1). Intake data were considered acceptable if both 85% of the feed supplied and 90% of the feed that disappeared from the bunk within the pen could be attributed to steers assigned to those bunks via radio frequency identification ear tag. During the experiment, all sampling days met these criteria; however, intake was not recorded in 6 d due to computer malfunctioning and these results were omitted from the analysis. Total BW gain (in kg) and total feed intake (in kg, DM basis) of each animal during the experiment were used for gain:feed ratio (**G:F**) calculation, and reported as g of BW gained per kg of DM consumed.

Blood samples were collected from all steers immediately after BW assessment on days 0, 28, 56, and 84. A final blood sample was also collected when steer BW was recorded the day before shipping to slaughter (days 112, 140, and 169; pre-slaughter sample). Blood was collected via jugular venipuncture into collection tubes (Vacutainer, 10 mL; Becton Dickinson, Franklin Lakes, NJ) containing freeze-dried sodium heparin. Blood samples were placed immediately on ice after collection, centrifuged (2,500 × *g* for 30 min; 4 °C) for plasma harvest, and stored at −20 °C on the same day of collection. Hot carcass weight was collected upon slaughter (days 114, 142, and 170). After a 24-h chill, trained personnel assessed carcass characteristics including backfat thickness at the 12th-rib, marbling, *Longissimus* muscle area, and presence of liver abscesses (Brown et al., 1975).

#### Rumen-cannulated heifers.

Individual feed intake was evaluated daily during each period using the GrowSafe 6000E software (GrowSafe System Ltd; Parsons et al., 2020). Intake data were considered acceptable as previously described for steers throughout each period, whereas intake was not recorded in 4 d due to computer malfunctioning. Heifers were sampled for blood and rumen fluid prior to TMR feeding (0700 h) on the first day of each period, and 4 h after TMR feeding (1200 h) on the final day of each period. Blood samples were collected as described for finishing steers. Whole rumen contents were extracted as in Cagle et al. (2020) using a suction strainer, and ruminal pH measured immediately after collection (Orion STAR A221 pH meter, Thermo Fisher Scientific, Waltham, MA). Rumen samples (approximately 200 mL) were strained through eight layers of cheesecloth for fluid extraction, which was stored into individual stainless-steel thermoses to maintain both temperature and an anaerobic environment and transported to the laboratory for further processing. One subsample (5 mL) of rumen fluid sample was transferred into falcon tubes containing 1 mL of metaphosphoric acid. Another subsample (2.5 mL) was transferred into falcon tubes containing 7.5 mL of metaphosphoric acid. Two other subsamples (10-mL each) were stored without any additives. All rumen fluid subsamples were stored at −20 °C on the same day of collection.

### Laboratorial Analyses

Feed samples were analyzed by wet chemistry procedures for concentrations of crude protein (method 984.13; AOAC, 2006), acid detergent fiber (method 973.18 modified for use in an Ankom 200 fiber analyzer, Ankom Technology Corp., Fairport, NY; AOAC, 2006), neutral detergent fiber using a-amylase and sodium sulfite (Van Soest et al., 1991; modified for use in an Ankom 200 fiber analyzer, Ankom Technology Corp.), starch (YSI 2700 SELECT Biochemistry Analyzer; YSI Inc., Yellow Springs, OH), ether extract (method 920.39; AOAC, 2006), and minerals using inductively coupled plasma emission spectroscopy (Sirois et al., 1991).

Plasma samples were analyzed in duplicates for concentrations of glucose and urea-N (respons910VET Veterinary Chemistry Analyzer; DiaSys Diagnostic Systems USA LLC, Wixom, MI; Rett et al., 2020), insulin (#PI-12K; EMD Millipore Corporation, Billerica, MA; Cooke et al., 2023), and insulin-like growth factor I (**IGF-I**; #SG100; R&D systems Inc., Minneapolis, MN; Cooke et al., 2012). Samples were re-analyzed if CV between duplicates was above 10% for all assays. The intra- and inter-assay CV for all these plasma analyses were <12%.

The 5-mL rumen fluid subsample with metaphosphoric acid was processed and analyzed for volatile fatty acid (**VFA**) profile (Cappellozza et al., 2013). The 2.5-mL rumen fluid subsample with metaphosphoric acid was analyzed for ammonia concentrations (Broderick and Kang, 1980). One of the 10-mL subsamples with no additive was analyzed for viscosity (SV-10/SV-100 Vibro Viscometer; A&D Company Ltd; Tokyo, Japan) as in Pitta et al. (2016), and the other subsample was analyzed for protozoa counts by a single technician as in Cagle et al. (2020) using a Sedgewick Rafter Counting Chamber (Hausser Scientific, Horsham, PA).

### Statistical Analysis

Data were analyzed as a 2 × 2 factorial design, with factor A being monensin + tylosin inclusion and factor B being *Y. schidigera* extract inclusion. Animal was considered the experimental unit for all analyses. Quantitative data were analyzed using the MIXED procedure of SAS (SAS Inst. Inc., Cary, NC), whereas binary data were analyzed using the GLIMMIX procedure of SAS (SAS Inst. Inc.) with a binomial distribution and logit link function. All data were analyzed using Satterthwaite approximation to determine the denominator degrees of freedom for tests of fixed effects. Data from finishing steers were analyzed using steer(factor A × factor B) as random variable, and data from rumen-cannulated heifers used heifer as random variable. Model statements for initial and final BW, ADG, feed efficiency, and carcass responses of finishing steers contained the effects of factor A, factor B, and the resultant interaction. Model statements for TMR intake and plasma variables of finishing steers contained the effects of factor A, factor B, day, and all resultant interactions. Plasma variables were analyzed using results from day 0 as an independent covariate, whereas TMR intake used baseline values (mean intake from days −7 to −1) as independent covariate. The specified term for these repeated statements was day, steer(factor A × factor B) was the subject, and the covariance structure used was first-order autoregressive based on the Akaike information criterion. The model statements for rumen and plasma variables from rumen-cannulated heifers contained the effects of factor A, factor B, the resultant interaction, in addition to period and results from the first day of each period as independent covariates. The model statement used for TMR intake of rumen-cannulated heifers contained the effects of factor A, factor B, day, all resultant interactions, and period as independent variable. The specified term for this repeated statement was day with heifer(factor A × factor B × period) as subject, and the covariance structure used was first-order autoregressive based on the Akaike information criterion. All results are reported as least square means, or covariately-adjusted least square means when model contained independent variables. Significance was set at *P* ≤ 0.05 and tendencies were determined if *P* > 0.05 and ≤ 0.10. Results are reported according to main factors (A or B) if no higher-order interactions containing factors were significant, or according to the highest-order interaction detected.

## Results

### Performance and Carcass Traits of Finishing Steers

No factor A × factor B interactions were detected these variables (*P* ≥ 0.32), and results are described according to each main factor (Tables 2 and 3). Steer BW and ADG were not impacted (*P* ≥ 0.88) while feed intake was decreased (*P* < 0.01) by inclusion of monensin + tylosin (Table 2). Hence, steer G:F during the experiment was increased (*P* = 0.02) by monensin + tylosin inclusion (Table 2). No effects of monensin + tylosin were noted (*P* ≥ 0.17) for carcass merit traits, including incidence of liver abscesses (Table 3). Inclusion of *Y. schidigera* extract did not impact (*P* ≥ 0.30) any of the performance and carcass traits evaluated herein (Tables 2 and 3).

**Table 2.** Performance responses of feedlot steers receiving diets containing (2 × 2 factorial arrangement): 1) monensin + tylosin (360 mg/steer daily from Rumensin 90 and 90 mg/steer daily from Tylan 40; Elanco Animal Health, Greenfield, IN) and 2) *Y. schidigera* extract (4 g/steer daily, as-fed basis; Micro-Aid Feed Grade Concentrate; DPI Global, Porterville, CA)^1^

Item	Monensin + tylosin (A)			*Y. schidigera* extract (B)			*P-values*		
	Yes	No	SEM	Yes	No	SEM	A	B	A × B
Average days on feed, d	165	165	—	165	165	—	—	—	—
BW parameters^2^									
Initial BW, kg	309	309	4	309	309	4	0.94	0.99	0.96
Final BW, kg	613	612	4	610	613	4	0.88	0.30	0.32
ADG, kg/d	1.83	1.82	0.03	1.82	1.83	0.03	0.83	0.83	0.25
Feed intake (dry matter),^3^ kg/d	11.5	12.2	0.11	11.9	11.9	0.11	<0.01	0.75	0.98
Gain to feed,^4^ kg/kg	0.155	0.145	0.003	0.148	0.149	0.003	0.02	0.62	0.82

^1^Steers were housed in four drylot pens equipped with electronic feed bunks (GrowSafe Systems Ltd, Airdrie, AB, Canada) that measured individual feed intake from day −14 to slaughter. Steers were slaughtered (STX Beef; Corpus Christi, TX) in three groups that contained an equivalent number of steers/treatment combination (36 steers on day 114, 36 steers on day 142, and 48 steers on day 170). Treatments were offered to cattle from day 0 to slaughter. Pens housed cattle from the same treatment combination, and cattle rotated across drylot pens every 14 d to account for any potential pen effect.

^2^Shrunk body weight (BW) recorded on day −16 (after 16-h feed and water restriction) was added a 4% shrink and considered initial BW. Final BW was calculated by averaging BW recorded the day of shipping to the packing plant and the day before. Average daily gain (ADG) was calculated using initial and final BW.

^3^Individual feed intake was computed daily using the GrowSafe 6000E software (GrowSafe Systems Ltd) as described by Parsons et al. (2020). Feed intake recorded from days −7 to 0 was averaged and included as independent covariate.

^4^Calculated using total BW gain (in kg) and total feed intake (in kg, dry matter) of each animal from day −16 to slaughter.

**Table 3.** Carcass parameters of feedlot steers receiving diets containing (2 × 2 factorial arrangement): 1) monensin + tylosin (360 mg/steer daily from Rumensin 90 and 90 mg/steer daily from Tylan 40; Elanco Animal Health, Greenfield, IN) and 2) *Y. schidigera* extract (4 g/steer daily, as-fed basis; Micro-Aid Feed Grade Concentrate; DPI Global, Porterville, CA)^1^

Item	Monensin + tylosin (A)			*Y. schidigera* extract (B)			*P-values*		
	Yes	No	SEM	Yes	No	SEM	A	B	A × B
Dressing, %	62.9	62.6	0.2	62.7	62.9	0.2	0.25	0.44	0.23
Hot carcass weight, kg	386	385	3	384	388	3	0.88	0.30	0.32
Backfat, cm	1.23	1.19	0.04	1.20	1.22	0.04	0.48	0.80	0.46
*Longissimus muscle* area, cm^2^	95.7	96.8	1.2	95.9	96.6	1.2	0.51	0.67	0.72
Marbling	449	433	11	446	437	11	0.30	0.55	0.77
Yield grade	2.71	2.59	0.07	2.65	2.65	0.07	0.28	0.96	0.82
Carcasses choice or prime, %	66.7	61.7	6.2	61.7	66.7	6.2	0.57	0.57	0.57
Incidence of liver abscess,^2^ %	1.67	6.66	2.6	3.33	5.00	2.6	0.17	0.65	0.65

^1^Steers were housed in four drylot pens equipped with electronic feed bunks (GrowSafe Systems Ltd, Airdrie, AB, Canada) that measured individual feed intake from day −14 to slaughter. Steers were slaughtered (STX Beef; Corpus Christi, TX) in three groups that contained an equivalent number of steers/treatment combination (36 steers on day 114, 36 steers on day 142, and 48 steers on day 170). Treatments were offered to cattle from day 0 to slaughter. Pens housed cattle from the same treatment combination, and cattle rotated across drylot pens every 14 d to account for any potential pen effect. Backfat thickness measured at the 12th rib; marbling score: 400 = Small^00^, 500 = Modest^00^; yield grade calculated as reported by Lawrence et al. (2010).

^2^Incidence of liver abscesses recorded as in Brown et al. (1975).

### Physiological Responses in Finishing Cattle

No factor A × factor B and factor A × factor B × day interactions (*P* ≥ 0.23), nor factor A × day and factor B × day interactions (*P* ≥ 0.17) were detected these variables. Hence, results are described according to main factors (Table 4). Mean concentrations of glucose, insulin, IGF-I, and urea-N were not affected (*P* ≥ 0.16) by monensin + tylosin nor by *Y. schidigera* extract inclusion.

**Table 4.** Plasma metabolites and hormones in feedlot steers receiving diets containing (2 × 2 factorial arrangement): 1) monensin + tylosin (360 mg/steer daily from Rumensin 90 and 90 mg/steer daily from Tylan 40; Elanco Animal Health, Greenfield, IN) and 2) *Y. schidigera* extract (4 g/steer daily, as-fed basis; Micro-Aid Feed Grade Concentrate; DPI Global, Porterville, CA)^1^^,^^2^

Item	Monensin + tylosin (A)			*Y. schidigera* extract (B)			*P-values*		
	Yes	No	SEM	Yes	No	SEM	A	B	A × B
Glucose, mg/dL	99.8	101	1	100	101	1	0.35	0.77	0.70
Insulin, ng/mL	18.6	17.7	0.9	17.6	18.8	0.9	0.47	0.35	0.23
Insulin-like growth factor I, ng/mL	168	172	5	170	171	5	0.55	0.83	0.55
Urea-N, mg/dL	14.5	14.0	0.3	14.0	14.3	0.3	0.16	0.40	0.74

^1^Steers were housed in four drylot pens equipped with electronic feed bunks (GrowSafe Systems Ltd, Airdrie, AB, Canada) that measured individual feed intake from day −14 to slaughter. Steers were slaughtered (STX Beef; Corpus Christi, TX) in three groups that contained an equivalent number of steers/treatment combination (36 steers on day 114, 36 steers on day 142, and 48 steers on day 170). Treatments were offered to cattle from day 0 to slaughter. Pens housed cattle from the same treatment combination, and cattle rotated across drylot pens every 14 d to account for any potential pen effect.

^2^Blood samples were collected from all steers prior to feeding on days 0, 28, 56, and 84. A final blood sample was also collected when steer body weight was recorded the day before shipping to slaughter (days 112, 140, and 169). Results from day 0 were included as independent covariate in each respective analysis. No interactions of factor with day were detected (*P* ≥ 0.23).

### Rumen-Cannulated Heifers

No interactions containing factor A, factor B, and day were detected (*P* ≥ 0.65) for feed intake in rumen-cannulated heifers, which was not impacted (*P* ≥ 0.42) by monensin + tylosin nor by *Y. schidigera* extract inclusion (reported according to main factors; Table 5). A factor A × factor B interaction was detected (*P* < 0.01) for ruminal ammonia concentrations. This variable was greater (*P* ≤ 0.05) in heifers that did not receive any of the feed additives compared with heifers that received *Y. schidigera* extract, monensin + tylosin, or a combination of all feed additives (2.09, 0.79, 0.81, and 1.27 mM, respectively; SEM = 0.29). Ruminal ammonia concentrations did not differ (*P* ≥ 0.23) among heifers that received the latter three treatment combinations.

**Table 5.** Feed intake and plasma variables in rumen-cannulated heifers receiving feedlot diets containing (2 × 2 factorial arrangement): 1) monensin + tylosin (360 mg/steer daily from Rumensin 90 and 90 mg/steer daily from Tylan 40; Elanco Animal Health, Greenfield, IN) and 2) *Y. schidigera* extract (4 g/steer daily, as-fed basis; Micro-Aid Feed Grade Concentrate; DPI Global, Porterville, CA)^1^^,^^2^

Item	Monensin + tylosin (A)			*Y. schidigera* extract (B)			*P-values*		
	Yes	No	SEM	Yes	No	SEM	A	B	A × B
Feed intake (dry matter),^3^ kg/d	12.1	12.9	0.6	12.6	12.5	0.6	0.42	0.89	0.99
Plasma variables									
Glucose, mg/dL	76.8	77.2	1.4	78.3	77.7	1.4	0.76	0.58	0.33
Insulin, ng/mL	34.0	38.1	5.5	33.7	38.4	5.5	0.50	0.42	0.82
Insulin-like growth factor I, ng/mL	77.7	75.6	6.5	75.6	77.7	6.5	0.73	0.73	0.52
Urea-N, mg/dL	17.5	15.8	0.9	16.0	17.3	0.9	0.21	0.32	0.88

^1^Heifers were assigned to a 4 × 4 Latin square design, containing four periods of 21 d each and a washout interval of 14 d between periods. During each 21-d period, heifers were housed in drylot pens equipped with electronic feed bunks (GrowSafe Systems Ltd, Airdrie, AB, Canada) that measured individual feed intake.

^2^Individual feed intake was computed daily using the GrowSafe 6000E software (GrowSafe Systems Ltd) as described by Parsons et al. (2020). Blood was sampled prior to feeding (0700 h) on the first day of each period, and 4 h after feeding (1200 h) on the final day of each period. Results obtained on the first day of each period were used as independent covariate in each respective analysis.

No other factor A × factor B interactions were detected (*P* ≥ 0.15) for ruminal responses, and these results are described according to main factors (Table 6). Rumen fluid pH was increased (*P* ≤ 0.04) by monensin + tylosin, and also by *Y. schidigera* extract inclusion (Table 6). Rumen fluid viscosity was reduced (*P* = 0.04) by *Y. schidigera* extract, and not affected (*P* = 0.65) by monensin + tylosin inclusion (Table 6). Rumen protozoa count was increased (*P* < 0.01) by monensin + tylosin, but was not altered (*P* = 0.56) by *Y. schidigera* extract inclusion (Table 6). Total rumen VFA concentrations were not affected (*P* ≥ 0.74) by monensin + tylosin nor by *Y. schidigera* extract inclusion (Table 6). Ruminal proportion of acetate, butyrate, iso-valerate, iso-butyrate, and valerate was also not impacted (*P* ≥ 0.16) by monensin + tylosin nor *Y. schidigera* extract inclusion (Table 6). The proportion of ruminal propionate was increased (*P* = 0.04) by monensin + tylosin, and tended (*P* = 0.07) to be increased by *Y. schidigera* extract inclusion (Table 6). Hence, ruminal acetate:propionate ratio was decreased (*P* = 0.03) by monensin + tylosin, and tended (*P* = 0.08) to be decreased by *Y. schidigera* extract inclusion (Table 6).

**Table 6.** Ruminal responses of rumen-cannulated heifers receiving feedlot diets containing (2 × 2 factorial arrangement): 1) monensin + tylosin (360 mg/steer daily from Rumensin 90 and 90 mg/steer daily from Tylan 40; Elanco Animal Health, Greenfield, IN) and 2) *Y. schidigera* extract (4 g/steer daily, as-fed basis; Micro-Aid Feed Grade Concentrate; DPI Global, Porterville, CA)^1^^,^^2^

Item	Monensin + tylosin (A)			*Y. schidigera* extract (B)			*P-values*		
	Yes	No	SEM	Yes	No	SEM	A	B	A × B
Rumen fluid ammonia, mM	1.04	1.44	0.2	1.03	1.45	0.2	0.18	0.15	< 0.01
Rumen protozoa count, 10^3^ per mL	812	387	78	572	627	77	<0.01	0.56	0.40
Rumen fluid pH	5.80	5.67	0.06	5.81	5.67	0.06	0.04	0.03	0.27
Rumen fluid viscosity, mPa.s.	13.8	19.7	9.9	3.1	29.9	10.0	0.65	0.04	0.80
Total rumen volatile fatty acids, mM	210	208	6	208	210	6	0.74	0.77	0.49
Acetate, mol/100 mol	49.6	51.4	1.1	49.7	51.3	1.1	0.16	0.23	0.15
Propionate, mol/100 mol	35.8	32.4	1.3	35.6	32.6	1.3	0.04	0.07	0.32
Butyrate, mol/100 mol	10.8	11.9	0.7	10.6	12.0	0.7	0.84	0.55	0.45
Iso-valerate, mol/100 mol	0.873	1.020	0.117	0.892	1.00	0.117	0.30	0.45	0.68
Iso-butyrate, mol/100 mol	0.581	0.591	0.029	0.573	0.599	0.029	0.80	0.52	0.88
Valerate, mol/100 mol	2.38	2.59	0.18	2.52	2.46	0.18	0.24	0.74	0.68
Acetate:propionate ratio	1.41	1.64	0.09	1.44	1.63	0.09	0.03	0.08	0.21

^1^Heifers were assigned to a 4 × 4 Latin square design, containing four periods of 21 d each and a washout interval of 14 d between periods. During each period, heifers were housed in drylot pens equipped with electronic feed bunks (GrowSafe Systems Ltd, Airdrie, AB, Canada) that measured individual feed intake.

^2^Rumen fluid was sampled prior to feeding (0700 h) on the first day of each period, and 4 h after feeding (1200 h) on the final day of each period. Rumen pH was measured using a pH meter concurrently with rumen fluid sampling. Rumen fluid samples were analyzed for viscosity assessment (Pitta et al., 2016), protozoa count (Caigle et al., 2020), ammonia (Broderick and Kang, 1980), and volatile fatty acid profile (Cappellozza et al., 2013). Results obtained on the first day of each period were used as independent covariate in each respective analysis.

## Discussion

Feedlot diets rich in fermentable carbohydrates often lead to digestive challenges and metabolic disorders in cattle (Nagaraja and Titgemeyer, 2007). Monensin and tylosin are traditionally incorporated into feedlot diets to inhibit bacteria that impair rumen and liver function (Amachawadi and Nagaraja, 2016). *Yucca schidigera* extract also inhibits growth of gram-positive bacteria and protozoa, and suggested as alternative or complement to monensin and tylosin (Cheeke, 2000). This experiment evaluated this latter rationale by using *Y. schidigera* extract (4 g/d; Rett et al., 2020) to replace or fed in conjunction with monensin + tylosin to feedlot cattle. No major interactive or complementary effects were observed with the combination of these additives, given the lack of factor A × factor B interactions in productive, physiological, and most of the ruminal responses. Hence, results are discussed according to main treatment factors but for ruminal ammonia concentrations, which yielded the only significant factor A × factor B interaction.

### Factor A (Monensin + Tylosin Inclusion)

Inclusion of monensin + tylosin into feedlot diets is known to reduce cattle feed intake while improving their feed efficiency (Heinemann et al., 1978; Stock et al., 1995; Callaway et al., 2003). These outcomes were noted in the present experiment, as feed intake decreased by 5.7% and G:F increased by 6.4% in steers that received monensin + tylosin. Steer BW gain and carcass merit traits, however, were not altered by monensin + tylosin inclusion. Others have also reported improved feed efficiency without changes in ADG when monensin + tylosin was added to feedlot diets (Potter et al., 1985; Stock et al., 1995; Marques and Cooke, 2021). The incidence of liver abscesses in this experiment was low compared with the latest average in U.S. feedlot cattle (17.8%; Eastwood et al., 2017), which may have hindered the proven benefits of tylosin. Nonetheless, incidence of liver abscesses was numerically decreased by 75% in finishing steers receiving monensin + tylosin, in accordance with values described in the literature (Potter et al., 1985; Nagaraja and Lechtenberg, 2007).

Plasma concentrations of glucose, insulin, and IGF-I are considered metabolic markers of energy intake and status in cattle (Yelich et al., 1995; Hess et al., 2005). The same rationale is applied to plasma urea-N concentrations with protein intake and metabolism (Hammond, 1997). Inclusion of monensin + tylosin did not impact these variables in finishing steers despite decreasing feed intake, suggesting improved dietary nutrient utilization (McGuffey et al., 2001; Weimer et al., 2008). These metabolic markers are also associated positively with BW gain in beef cattle (Ellenberger et al., 1989; Hersom et al., 2004), which corroborates the similar ADG of steers receiving or not monensin + tylosin.

Rumen-cannulated heifers were assigned to an experimental design that focused on ruminal responses rather than performance measurements. Nonetheless, their feed intake was also numerically reduced by 5.7% when monensin + tylosin was added to their diets. Inclusion of monensin + tylosin increased ruminal pH and shifted ruminal fermentation toward propionate production, as well-established in the literature (Callaway et al., 2003; Nagaraja and Lechtenberg, 2007). Monensin also improves dietary protein utilization by mitigating ruminal proteolysis, and stimulates the somatotropic axis by increasing propionate availability for glucose synthesis (Goodrich et al., 1984; Huntington, 1997; Rogers et al., 1997). Inclusion of monensin + tylosin sustained plasma concentrations of glucose, insulin, IGF-I, and urea-N in heifers despite the numerical reduction in feed intake, suggesting improved dietary nutrient utilization as noted for finishing steers (McGuffey et al., 2001; Weimer et al., 2008).

Protozoa count in the rumen nearly doubled when monensin + tylosin was added to the diet of rumen-cannulated heifers. Purser et al. (1965) reported that tylosin supplementation increased protozoa count in the rumen of sheep by 70%, although research investigating the individual ruminal role of tylosin is limited. In contrast, monensin can reduce the rumen protozoa population via similar mechanisms by which inhibits bacterial growth (Dennis and Nagaraja, 1986; Rogers et al., 1997; Guan et al., 2006). Research that evaluated monensin and tylosin fed in conjunction, however, did not observe increased protozoa count in grain-fed cattle (Ives et al., 2002; Shen et al., 2018). Rumen protozoa are known to adapt and build resistance to antimicrobials including ionophores, particularly when added to high-concentrate diets (Dennis and Nagaraja, 1986; Guan et al., 2006). Heifers used herein did not receive monensin nor tylosin prior to the beginning of the experiment, and the 14-d washout interval between experimental periods is longer than the lifespan of rumen protozoal species (Jouany et al., 1988; Williams and Coleman, 1997). Ruminal protozoa are also associated positively with ruminal fluid viscosity and ammonia content (Mangan, 1959; Clarke, 1965; Jouany, 1996), and negatively with propionate synthesis (Williams and Coleman, 1997; Newbold et al., 2015). Dietary inclusion of monensin + tylosin did not increase viscosity or ammonia concentrations in the rumen fluid, and favored propionate synthesis as previously described. Hence, the reasons why inclusion of monensin + tylosin increased rumen protozoa in this experiment is unknown, despite not yielding negative consequences to rumen fermentation responses.

### Factor B (Y. schidigera Extract Inclusion)

*Yucca schidigera* extract has been associated with improved ruminal digestibility in forage-fed steers (McMurphy et al., 2014a), and performance responses of cattle consuming high-grain diets (Sousa et al., 2019; Rett et al., 2020). More specifically, McMurphy et al. (2014a) reported that supplementing *Y. schidigera* extract at 2 g/d improved *in situ* rumen DM and neutral detergent fiber digestibility in beef steers, but without affecting their feed intake. Sousa et al. (2019) supplemented *Y. schidigera* extract at 2 g/d to high-risk cattle during a 60-d feedlot-receiving period, and observed increased ADG and feed efficiency with no impacts to feed intake. Authors credited these benefits to improved rumen fermentation conditions, particularly increased ruminal propionate production. Accordingly, Rett et al (2020) reported that supplementing *Y. schidigera* extract at 4 g/daily to beef heifers receiving a grain-based bloat provocative diet yielded the greatest improvements in ADG and feed efficiency, and also favored rumen propionate concentration. In the present experiment, feed intake was not altered as in McMurphy et al. (2014a) and Sousa et al. (2019), but no performance benefits were observed when *Y. schidigera* extract was provided to finishing steers. McMurphy et al. (2014b) also reported that ADG was not improved in stocker cattle receiving *Y. schidigera* extract at 2 g/d during an 85-d grazing period. Both Sousa et al. (2019) and Rett et al. (2020) evaluated *Y. schidigera* extract supplementation in high-stress experimental models, which was not in McMurphy et al. (2014b) and this experiment. The *Y. schidigera* extract has been associated positively with cattle immunocompetence due to its saponin content (Cheeke, 2000), and the high-risk steers receiving *Y. schidigera* extract in Sousa et al. (2019) required fewer antimicrobial treatments to recover from respiratory disease. Perhaps the performance benefits from *Y. schidigera* extract are mainly manifested in beef cattle exposed to high-stress, immunocompromising conditions as in Sousa et al. (2019) and Rett et al. (2020).

Inclusion of *Y. schidigera* extract did not improve any of the carcass merit traits, nor impacted the incidence of liver abscesses in finishing steers. Wallace et al. (1994) characterized the antibacterial effects of *Y. schidigera* extract in rumen fluid cultures, including suppression of *Streptococcus* spp. that are associated with liver abscesses. Therefore, *Y. schidigera* extract may be an alternative to mitigate incidence of liver abscesses in feedlot cattle, but the low prevalence of this condition herein hindered such evaluation. Plasma concentrations of glucose, insulin, IGF-I, and urea-N in finishing steers were also not impacted by dietary inclusion of *Y. schidigera* extract, in agreement with feed intake, feed efficiency, and ADG results (Ellenberger et al., 1989; Hersom et al., 2004; Hess et al., 2005). Likewise, *Y. schidigera* extract did not impact feed intake and plasma concentrations of these metabolic markers in rumen-cannulated heifers.

*Yucca schidigera* extract also suppresses the growth of gram-positive bacteria that cause ruminal acidosis such as *S. bovis* (Wallace et al., 1994), which helps explaining the greater ruminal pH in rumen-cannulated heifers that received *Y. schidigera* extract. McMurphy et al. (2014a) and Rett al. (2020) did not report changes in rumen pH when supplementing *Y. schidigera* extract, but these authors did not evaluate typical corn-based finishing diets. Despite its surfactant properties and potential to yield foaming effects (Cheeke, 2000), supplementing *Y. schidigera* extract reduced rumen fluid viscosity in rumen-cannulated heifers. This outcome has direct implications to frothy bloat (Cheng et al., 1998; Pitta et al., 2016), although no incidence of this digestive disorder was noted in this experiment. Rett et al. (2020) supplemented *Y. schidigera* extract to mitigate frothy bloat in grain-fed cattle, and reported that heifers receiving 0 or 4 g/d of *Y. schidigera* extract had equivalent rumen fluid viscosity. Rett et al. (2020) fed a bloat-provocative diet (Bartley et al., 1983) that may have altered ruminal environment to an extent that nullified some of the benefits from *Y. schidigera* extract. Therefore, results from this experiment support the potential of *Y. schidigera* extract in preventing excessive rumen fluid viscosity and subsequent risk of bloat in feedlot cattle (Cheng et al., 1998; Pitta et al., 2016).

Dietary inclusion of *Y. schidigera* extract did not reduce rumen protozoa count in rumen-cannulated heifers, despite its antiprotozoal effects reported by others (Cheeke, 2000; McMurphy et al., 2014a). Rett et al. (2020) noted similar rumen protozoa count in heifers receiving a bloat-provocative diet with or without the inclusion of *Y. schidigera* extract at 4 g/heifer daily. Benchaar et al. (2008) supplemented *Y. schidigera* extract to lactating dairy cows consuming a high-grain diet, and did not observe any reductions in ruminal protozoa count as total numbers or according to specific genera. Grain-based diets with high fermentative capacity favor the proliferation of protozoa in the rumen (Williams and Coleman, 1997), which may limit the antiprotozoal effects of *Y. schidigera* extract as suggested by Rett et al. (2020). In turn, dietary inclusion of *Y. schidigera* extract favored ruminal propionate synthesis without impacting total VFA production in rumen-cannulated heifers, as reported by Rett et al. (2020) and others (Hristov et al., 1999; Holtshausen et al., 2009). *Yucca schidigera* extract inhibits growth of rumen bacterial species not involved in propionate synthesis such as *S. bovis* and *Butyrivibrio fibrisolvens* (Wallace et al., 1994), facilitating the proliferation of propionate-producing bacteria (Hristov et al., 1999). *Yucca schidigera* extract can also increase valerate and iso-valerate concentrations in the rumen (Holtshausen et al., 2009; Rett al., 2020), but these VFA were not affected by *Y. schidigera* extract inclusion in this experiment as also observed by others (Hristov et al., 1999; Santoso et al., 2004)

### Factor A × B Interaction

*Yucca schidigera* extract and ionophores including monensin have been shown to decrease ruminal proteolysis, resulting in less accumulation of ammonia in the rumen (Yang and Russell, 1993; Wallace et al., 1994). Accordingly, ruminal ammonia concentration in rumen-cannulated heifers was decreased by dietary inclusion of *Y. schidigera* extract and monensin + tylosin, although no complimentary effects were observed by the combination of these feed additives. These outcomes may have contributed to improved dietary protein utilization and subsequent feed efficiency in cattle receiving monensin + tylosin, but was not sufficient to yield similar effects in cattle that received *Y. schidigera* extract.

### Overall Conclusions

Inclusion of *Y*. *schidigera* extract into a finishing diet modulated ruminal pH and shifted ruminal fermentation toward propionate synthesis in a similar manner as monensin + tylosin inclusion. Rumen fluid viscosity was also reduced with *Y. schidigera* extract, demonstrating its potential in mitigating bloat in feedlot cattle. The increased feed efficiency of finishing steers that received monensin + tylosin, which has been well-established in the literature, was not observed with *Y. schidigera* extract inclusion. No complimentary effects were observed in ruminal, physiological, and productive responses when combining all these additives into the finishing diet. Nonetheless, results from this experiment corroborate the benefits of *Y. schidigera* extract to ruminal environment and fermentation in feedlot cattle, which were not sufficient to improve the physiological and productive responses evaluated herein.
